# Book Review of South African Family Practice Manual: 4th edition

**DOI:** 10.4102/phcfm.v15i1.4207

**Published:** 2023-07-27

**Authors:** Steve Reid

**Affiliations:** 1 Department of Family, Community and Emergency Care, Primary Health Care Directorate, Faculty of Health Sciences, University of Cape Town, Cape Town, South Africa

This revised edition of the *South African Family Practice Manual*, first published in 1995, is a huge contribution to the field of family practice not only in South Africa but also in many middle- and low-income countries particularly in Africa. The editor Bob Mash has widened his editorial team to manage a remarkable 194 chapters, covering a vast array of practical skills. The range of topics covered by the chapters illustrates the extremely wide scope of practice demanded of generalists, which goes far beyond the manual skills of specific procedures, including: (1) communication, (2) working with interpreters, (3) engaging with communities, (4) teaching and dealing with resilience and (5) burnout, for example. So, as far as comprehensiveness is concerned, the manual is impressive and clearly delineates the role of the generalist practitioner in the South African context.

The list of authors is extremely diverse, with not only family physicians and general practitioners but also specialists from a wide variety of disciplines, including public health, anaesthetics, even plastic surgery and radiation oncology. Many of the emergency skills lie squarely in the domain of the developing specialty of Emergency Medicine, with which Family Medicine has a close relationship in South Africa, and which is fast developing in other countries. While there needs to be overlap, the differentiation of the specialties is important, and this manual helps to specify the skills that are in common as well as those that are more appropriate to generalist practice.

Despite the chapters on family and community engagement, the broad framing of the skills required of the generalist medical practitioner is still too biomedical and curative oriented, with not enough emphasis on health promotion and how to address the social determinants of health that drive the burden of disease. The only issue that, the author feels, does not receive appropriate attention is that of leadership, teamwork and referrals to other levels of care, although it is mentioned as important in several chapters, particularly the community-oriented and rehabilitation chapters. Tackling the social determinants of health demands a structured team approach, and the role of the medical practitioner needs to be negotiated, as it is not necessarily that of the leader, especially at the community level. How best to contribute to the wider systems that concern health, is a high-level skill required of the medical practitioner that possibly requires a separate chapter in the future.

The book is very well laid out, with clear writing and extensive diagrams and images in most chapters. The chapters are succinct and to the point, to the extent of being not detailed enough in some instances, where other resources would need to be consulted before attempting a particular procedure (e.g. Chapter 46 – How to do a Caesarian section). Nevertheless, most chapters offer a good introduction to each procedure, and the bibliography at the end of the book gives some guidance to further reading. This could be enhanced by the inclusion of two or three key references at the end of each chapter, for those interested in taking it further.

The manual deserves to be on the desk of every career generalist practitioner in Africa, from clinical associates or officers, to medical officers, general practitioners and family physicians. Particularly in rural hospitals and health centres, where specialist advice or supervision is not immediately available, the manual fulfils the crucial function of giving practitioners an entry point into the wide range of practical skills demanded of the generalist, which at first appears to be overwhelming to the young community service officer. Having this book at each district hospital in the country should go a long way to mitigating that anxiety.

